# Epidemiology of *Escherichia coli* O157:H7 Outbreaks, United States, 1982–2002

**DOI:** 10.3201/eid1104.040739

**Published:** 2005-04

**Authors:** Josefa M. Rangel, Phyllis H. Sparling, Collen Crowe, Patricia M. Griffin, David L. Swerdlow

**Affiliations:** * Centers for Disease Control and Prevention, Atlanta, Georgia, USA;; † Cincinnati Children's Hospital Medical Center, Cincinnati, Ohio, USA;; ‡ U.S. Department of Agriculture, Athens, Georgia, USA

**Keywords:** Escherichia coli O157:H7, disease outbreaks, population surveillance, United States/epidemiology, food, human, Centers for Disease Control and Prevention (U.S.), research

## Abstract

*Escherichia coli* O157:H7 causes 73,000 illnesses in the United States annually. We reviewed *E. coli* O157 outbreaks reported to Centers for Disease Control and Prevention (CDC) to better understand the epidemiology of *E. coli* O157. *E. coli* O157 outbreaks (≥2 cases of *E. coli* O157 infection with a common epidemiologic exposure) reported to CDC from 1982 to 2002 were reviewed. In that period, 49 states reported 350 outbreaks, representing 8,598 cases, 1,493 (17%) hospitalizations, 354 (4%) hemolytic uremic syndrome cases, and 40 (0.5%) deaths. Transmission route for 183 (52%) was foodborne, 74 (21%) unknown, 50 (14%) person-to-person, 31 (9%) waterborne, 11 (3%) animal contact, and 1 (0.3%) laboratory-related. The food vehicle for 75 (41%) foodborne outbreaks was ground beef, and for 38 (21%) outbreaks, produce.

## Abstract 2

Surveillance data from 350 U.S. outbreaks of *Escherichia coli* O157:H7 are analyzed.

*Escherichia coli* O157:H7 was first recognized as a pathogen in 1982 during an outbreak investigation of hemorrhagic colitis (1). *E. coli* O157 infection can lead to hemolytic uremic syndrome (HUS), characterized by hemolytic anemia, thrombocytopenia, and renal injury (2). Still, it was not until 1993, after a large multistate *E. coli* O157 outbreak linked to undercooked ground beef patties sold from a fast-food restaurant chain (3), that *E. coli* O157 became broadly recognized as an important and threatening pathogen. Clinical laboratories began examining more stool specimens for *E. coli* O157 (4). In 1994, *E. coli* O157 became a nationally notifiable infection, and by 2000, reporting was mandatory in 48 states. An estimated 73,480 illnesses due to *E. coli* O157 infection occur each year in the United States, leading to an estimated 2,168 hospitalizations and 61 deaths annually (5), and it is an important cause of acute renal failure in children (6,7).

Although reported outbreaks account for only a minority of *E. coli* O157 cases, outbreak investigations contribute greatly to understanding *E. coli* O157 epidemiology by identifying transmission routes, vehicles, and mechanisms of contamination (8). Outbreak findings oblige regulatory and public health agencies and industry to evaluate prevention and control measures so similar outbreaks can be prevented. Knowledge of transmission routes and vehicles allows consumers to be educated on reducing risky behavior that can decrease their risk for infection. We report here surveillance results for *E. coli* O157 outbreaks reported to the Centers for Disease Control and Prevention (CDC) from 1982 through 2002, to highlight the epidemiology of this emerging pathogen.

## Methods

Outbreaks of *E. coli* O157:H7 and Shiga toxin–producing *E. coli* O157:NM (subsequently referred to as *E. coli* O157) investigated by state and local health departments were reported to CDC by telephone, outbreak report, or through the routine foodborne disease outbreak surveillance system (9). In preparation for this summary, an epidemiologist reviewed all reports including published outbreaks not otherwise reported. Information collected from each outbreak report included city, setting, and suspected transmission route and vehicle. The date of first illness, hospitalizations, number of ill persons, bloody diarrhea, culture-confirmed illness, HUS, and deaths were also obtained. We defined an outbreak as ≥2 cases of *E. coli* O157 infection (at least 1 culture-confirmed) with a common epidemiologic exposure. For purposes of defining an outbreak, we considered a case as a stool culture yielding *E. coli* O157, or bloody diarrhea, or HUS. Each investigator reported the total number of outbreak-related cases, often including those with compatible clinical illness but without culture confirmation of illness. Infections acquired outside the United States were not included.

We defined outbreak onset as month and year first illness onset was reported, and outbreak setting as place where exposure occurred. Outbreaks due to a distributed food item and not isolated to a single venue or event were classified as communitywide. Fast-food settings were defined as establishments where payment is made before receiving food. Outbreaks were classified into 1 of 6 transmission routes on the basis of how most patients acquired the infection (foodborne, person-to-person, recreational water, drinking water, animal exposure, or laboratory). Outbreaks with a common exposure but in which a major transmission route was not identified were classified as unknown transmission route. Median outbreak sizes were compared by using the Kruskal-Wallis test. Outbreak-related HUS and death rates were compared by using a chi-square test.

Foodborne outbreaks were defined as the occurrence of ≥2 cases of *E. coli* O157 infection resulting from ingestion of a common food, or if food vehicle was undetermined, sharing a common meal or food facility. Food vehicles were grouped into the following categories: ground beef, other beef, produce, dairy, other, or unknown. Food vehicles were implicated statistically in case-control studies (p ≤ 0.05), by isolation of *E. coli* O157 from a suspect item, or by being the only common food item consumed by cases. A multistate outbreak was defined as exposure to a common vehicle occurring in >1 state. HUS cases were classified by individual investigators and included those cases diagnosed as thrombotic thromobocytopenic purpura following *E. coli* O157 infection.

## Results

**Figure 1:**
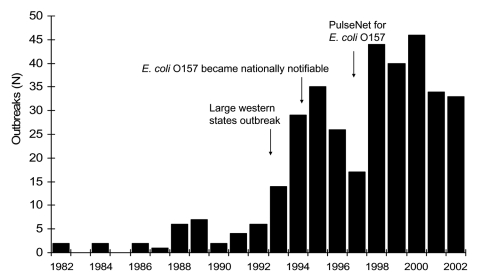
*Escherichia coli* O157 outbreaks by year, 1982–2002 (N = 350).

**Figure 2:**
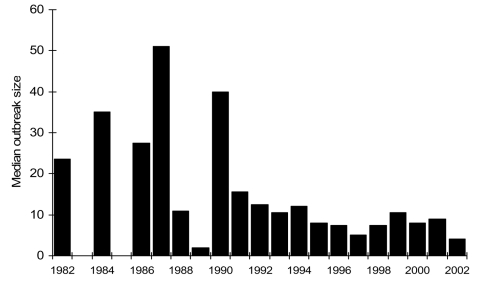
Median size of *Escherichia coli* O157 outbreaks by year.

From 1982 to 2002, a total of 350 outbreaks were reported from 49 states, accounting for 8,598 cases of *E. coli* O157 infection. Among cases, there were 1,493 (17.4%) hospitalizations, 354 (4.1%) cases of HUS, and 40 (0.5%) deaths. The number of reported outbreaks began rising in 1993, and peaked in 2000 with 46 (Figure 1). Outbreak size ranged from 2 to 781 cases, with a median of 8 cases. Median outbreak size appears to have declined from 1982 to 2002 (Figure 2). Most outbreaks (89%) occurred from May to November. Of the 326 outbreaks reported from a single state, Minnesota reported the most (43 outbreaks), followed by Washington (27 outbreaks), New York (22 outbreaks), California (18 outbreaks), and Oregon (18 outbreaks). Among the 350 outbreaks, transmission routes for 183 (52%) were foodborne, 74 (21%) unknown, 50 (14%) person-to-person, 21 (6%) recreational water, 11 (3%) animal contact, 10 (3%) drinking water, and 1 (0.3%) laboratory-related transmission route (Table ).

**Table:** Outbreaks and cases of *Escherichia coli* O157 infection by transmission route, 1982–2002

Transmission route	Outbreaks			Outbreak size	Cases		
	n	Total %	Foodborne %	Median (range)	n	Total %	Foodborne %
Ground beef	75	21	41	8 (2–732)	1,760	20	33
Unknown food vehicle	42	12	23	8 (2–86)	646	8	12
Produce	38	11	21	20 (2–736)	1,794	21	34
Other beef	11	3	6	17 (2–323)	563	7	11
Other food vehicle	10	3	5	15 (2–47)	206	2	4
Dairy product	7	2	4	8 (2–202)	300	3	6
Subtotal, foodborne	183	52		11 (2–736)	5,269	61	
Unknown transmission route	74	21		4 (2–140)	812	9	
Person-to-person	50	14		7 (2–63)	651	8	
Recreational water	21	6		8 (2–45)	280	3	
Animal contact	11	3		5 (2–111)	319	4	
Drinking water	10	3		26 (2–781)	1,265	15	
Laboratory-related	1	<1		2	2	<1	
Subtotal, other routes	167	48		5 (2–781)	3,329	39	
Total	350				8,598		

### Foodborne Outbreaks

**Figure 3:**
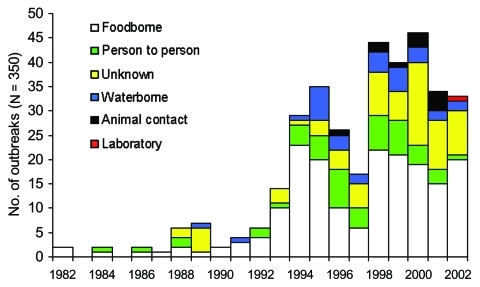
Transmission routes of *Escherichia coli* O157 outbreaks by year.

Food remained the predominant transmission route from 1982 to 2002 (Figure 3), accounting for 52% of 350 outbreaks and 61% of 8,598 outbreak-related cases. Foodborne outbreaks most frequently occurred in communities (53 [29%] of 183), restaurants/food facilities (51 [28%]), and schools (16 [9%]). Median size of foodborne outbreaks varied by setting: the smallest occurred in individual residences (3 cases), and the largest outbreaks in residential facilities (44 cases), followed by camps (36 cases). Among 51 restaurant and food facility outbreaks, 22 were in chain establishments (including 12 fast-food establishments) and 29 in single establishments. The median number of cases per restaurant/food facility outbreak was larger in chain than single establishments (21 vs. 8, p < 0.001). Among the 183 foodborne outbreaks, the food vehicle in 75 (41%) was ground beef, in 42 (23%) was unknown, in 38 (21%) was produce, in 11 (6%) was other beef, in 10 (5%) was other foods, and in 7 (4%) was dairy products.

### Ground Beef

**Figure 4:**
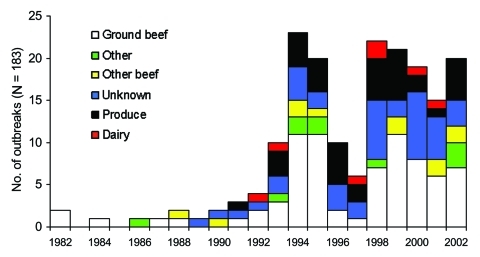
Vehicles of foodborne *Escherichia coli* O157 outbreaks by year.

The first *E. coli* O157 outbreak was reported in 1982 and linked to ground beef, which remains the most common vehicle among foodborne outbreaks (75 [41%] of 183) (Figure 4), although it accounts for only 33% of 5,269 foodborne-related cases. Outbreaks involving ground beef peaked in summer months: 71% occurred from May to August. Of the 40 outbreaks for which ground beef preparation style was reported, 27 (68%) were linked to hamburgers and 5 (13%) to meat sauce. Ground beef–associated outbreaks occurred most frequently at the communitywide level (36 of 75 [48%]), followed by 11 (15%) at picnics or camps, 8 (11%) at individual residences, 7 (9%) at restaurants, and 4 (5%) at schools. Of the 7 ground beef–associated restaurant outbreaks, 5 occurred in fast-food restaurants in 1982 (2 outbreaks), 1992–1993 (1 outbreak), 1995 (1 outbreak), and 1999 (1 outbreak). The last hamburger-associated fast-food restaurant outbreak was reported in 1995.

### Other Beef

Types of beef other than ground beef were implicated in 11 outbreaks. Five outbreaks were associated with consumption of roast beef, 2 with steak, 1 with sirloin tips, and 1 with salami. The other 2 outbreaks were identified only as "beef" and "raw roast beef."

### Produce

Produce-associated outbreaks were first reported in 1991 and have remained a prominent food vehicle (Figure 4), accounting for 38 (21%) of 183 foodborne outbreaks and 34% of 5,269 foodborne outbreak-related cases. Produce-associated outbreaks peaked in summer and fall; 74% occurred from July to October. Thirteen (34%) produce outbreaks were from lettuce, 7 (18%) from apple cider or apple juice, 6 (16%) from salad, 4 (11%) from coleslaw, 4 (11%) from melons, 3 (8%) from sprouts, and 1 (3%) from grapes. Produce-associated outbreaks most commonly occurred in restaurants (15 [39%]), and 7 (47%) of these were reported to be due to cross-contamination during food preparation. Twenty (53%) produce-associated outbreaks did not involve kitchen-level cross-contamination, including the 7 outbreaks associated with apple cider or apple juice, 7 of 10 lettuce-associated outbreaks, 3 of 4 coleslaw-associated outbreaks, and the 3 alfalfa-associated or clover sprout-associated outbreaks. None were reported to be due to imported produce. The median number of cases in produce-associated outbreaks was significantly larger than that of ground beef-associated outbreaks, 20 vs. 8, (p < 0.001).

### Dairy Products

Seven outbreaks were associated with dairy products, including 4 from consuming raw milk. The others were due to cheese curds made from raw milk, from butter made from raw milk, and from commercial ice cream bars (possibly due to cross-contamination).

### Person-to-Person Outbreaks

Fifty outbreaks were spread by the fecal-oral route. Outbreak settings included 40 (80%) child daycare centers; 5 (10%) individual residences; 3 (6%) communities, 1 (2%) school, and 1 (2%) residential facility. Outbreak size ranged from 2 to 63 cases (median 7). Person-to-person outbreaks peaked during summer; 70% occurred from June to August.

### Waterborne Outbreaks

Thirty-one outbreaks were waterborne: 21 from recreational water and 10 from drinking water. Recreational water-associated outbreaks were first reported in 1991; 14 (67%) occurred in lakes or ponds, and 7 (33%) in swimming pools. Outbreak size ranged from 2 to 45 cases (median 8 cases). Outbreaks occurred from June to September.

Outbreaks due to contaminated drinking water tended to be much larger than all other outbreaks, with a median size of 26 vs. 8 cases, (p = 0.08) and occurred from May to December. Drinking water outbreaks accounted for 3% of all outbreaks, but 15% of all outbreak-related cases. Four of the outbreaks were attributed to local well water systems, 3 involved municipal water supply systems, and 1 each was due to spring water, residential faucet water, and ice thought to be cross-contaminated. Two of the 3 municipal water suppliers did not use chlorination, and the other had a malfunctioning chlorinator.

### Animal Contact Outbreaks

First reported in the United States in 1996, outbreaks due to animal contact are 1 of the newest recognized transmission routes. Direct or indirect cow or calf exposure was noted in all 11 outbreaks: 5 on farms, 2 at county fairs, 2 at petting zoos, 1 at a barn dance, and 1 at a camp. Nine of the outbreaks occurred from July to November. Outbreak size ranged from 2 to 111 cases and accounted for 4% of the 8,598 outbreak-related cases.

### Laboratory-related Outbreak

One outbreak was reported in 2002 from a laboratory. It involved 2 culture-confirmed cases. Two technicians were infected while validating an *E. coli* O157 sterilization technique.

### Outbreaks with Unknown Transmission Route

Outbreaks reported as unknown transmission route accounted for 21% of outbreaks and 9% of all outbreak-related cases. Most (92%) occurred from May to November. Median size was 4 cases (range 2–140).

### Multistate Outbreaks

Twenty-four multistate *E. coli* O157 outbreaks were reported since 1992; they ranged from 1 to 3 per year, except in 1999, when 6 were reported. The number of states involved ranged from 2 to 8 with a median of 3. All were due to foodborne transmission. Sixteen (67%) were from ground beef and 6 (25%) from produce.

### HUS Cases

**Figure 5:**
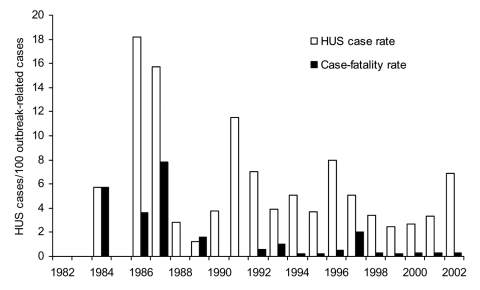
Hemolytic uremic syndrome (HUS) and case-fatality rate per 100 outbreak-related illnesses.

Among 346 outbreaks that reported HUS cases, 132 (38%) reported at least 1 case of HUS (range 1–55 cases, median 2 cases), for a total of 354 HUS cases. The HUS rate (number of cases per 100 outbreak-related illnesses) was 4.1. From 1982 to 2002, the HUS rate appeared to decline overall (Figure 5). The HUS rate differed significantly by transmission route (p < 0.001) and was highest among swimming outbreaks (10.7), followed by person-to-person (6.8), unknown (6.7), animal contact (5.6), foodborne (3.5), and drinking water (2.1) related–outbreaks. Among foodborne outbreaks, the HUS case rate was significantly higher among ground beef–associated outbreaks compared with all other foodborne outbreaks (5.5 vs. 2.5, p < 0.001).

### Deaths

Among 325 outbreaks that reported number of deaths, 25 (8%) reported at least 1 (range 1–4), for a total of 40 deaths. Twenty-five (63%) deaths were in persons with HUS; 15 (38%) were due to other causes. Among 12 outbreaks reporting age at death, age ranges were 1–4 years and 61–91 years. Case-fatality rate (number of deaths per 100 outbreak-related illnesses) was 0.5 and appeared to decrease from 1982 to 2002 (Figure 5). The case-fatality rate did not vary significantly by transmission route; however, the rate was significantly higher among outbreaks in residential facilities than in other settings (6.6 vs. 0.4, p < 0.001). Residential facilities where deaths occurred included a nursing home, a custodial institution, and an acute-care facility.

## Discussion

From 1982 to 2002, a total of 350 *E. coli* O157 outbreaks were reported in the United States from 49 states. Despite regulatory efforts to improve the safety of the U.S. food supply, foodborne *E. coli* O157 outbreaks remain common. Ground beef remains the most frequently identified vehicle, and produce-associated outbreaks are commonly reported. In addition, nonfoodborne transmission routes remain prominent. Person-to-person outbreaks occur most frequently in child daycare centers. Waterborne outbreaks caused by both drinking and recreational water continue to be reported, and outbreaks due to animal contact are increasingly reported.

In January 1993, the largest *E. coli* O157 outbreak from ground beef was reported in 4 western states, involving >700 ill persons, mostly children; more than one quarter were hospitalized, HUS developed in 7.5%, and 4 children died (3,10). Illness was linked to eating undercooked hamburgers at a chain fast-food restaurant, prompting a recall of >250,000 hamburgers, which likely prevented many additional illnesses and deaths.

Outbreak investigations that implicated fast-food hamburgers have led to major improvements in meat safety in the U.S fast-food industry. In 1993, the U.S. Food and Drug Administration revised the Model Food Code for restaurants, with new temperature guidelines for ground beef (11). In 1994, the National Livestock and Meat Board's Blue Ribbon Task Force developed objective measures of meat "doneness" and encouraged use of automated cooking systems (12). No fast-food hamburger-associated outbreaks have been reported since 1995, demonstrating that changes in the fast-food industry, such as carefully regulating cooking temperature of hamburgers, are both possible and effective.

In addition, outbreak investigations coupled with traceback investigations of implicated meat have identified contaminated beef lots, leading to large recalls of potentially contaminated beef (3). These recalls of up to 25 million pounds of beef (13) likely prevented many additional infections. Despite these improvements, ground beef continues to be frequently implicated in *E. coli* O157 outbreaks. Raw beef, especially ground beef, can be contaminated with *E. coli* O157 and should be cooked thoroughly to kill pathogens and handled carefully to avoid cross-contamination of other food items. As ground beef outbreaks are commonly reported from home-prepared ground beef, educational efforts should be focused on teaching consumers safer handling and cooking practices.

Outbreaks provide information about inadequacy of processing methods. For example, in 1994, an *E. coli* O157 outbreak due to eating commercially distributed dry-cured salami product involved 23 persons; HUS developed in 13% (14). This outbreak prompted U.S. Department of Agriculture officials to develop regulations to ensure the safety of shelf-stable fermented sausages (15); no further *E. coli* O157 outbreaks due to U.S.–manufactured salami have been reported since.

*E. coli* O157 outbreaks due to produce have become increasingly common. While half of produce-associated outbreaks were due to kitchen-level cross-contamination, which calls for further prevention efforts targeting food preparers, the other half were due to produce already contaminated with *E. coli* O157 before purchase, including lettuce, sprouts, cabbage, apple cider, and apple juice (16–20). These produce items could have become contaminated in the field from manure or contaminated irrigation water; during processing due to contaminated equipment, wash water, or ice or poor handling practices; during transport; or through contaminated storage equipment. Washing produce with water or a chlorine-based solution reduces *E. coli* O157 counts only modestly (21,22); therefore, once consumers obtain contaminated produce intended for raw consumption, little can be done to prevent illness. Efforts by industry to decrease contamination of sprouts have had limited success (23,24). Until effective measures for preventing *E. coli* O157 contamination of produce items such as lettuce, cabbage, and sprouts can be implemented, consumers should be educated about potential risk of consuming these items raw. Further regulatory and educational efforts are needed to improve the safety of produce items.

In 1996, a large *E. coli* O157 outbreak occurred in 3 western states and British Columbia, involving 70 illnesses, mostly children; more than one third of patients were hospitalized, HUS developed in 20%, and 1 child died (20). Illness was attributable to drinking commercial unpasteurized apple juice. However, as a result of this outbreak investigation, apple cider and apple juice that are shipped interstate in the United States since 1998 are either pasteurized or, if sold raw, carry a warning label advising consumers of potential harmful bacteria in the product (25). Since 1998, only 2 outbreaks due to unpasteurized apple cider have been reported, 1 at a local fair and 1 from locally produced cider that carried a warning label.

Prevention efforts focused on hygiene are needed to reduce transmission in daycare settings. In outbreaks of other primary transmission routes, secondary cases occur, which emphasizes the importance of educating caretakers to avoid direct contact with fecal matter and to apply stringent handwashing rules.

Drinking and recreational water have the potential to infect many persons. The largest U.S. *E. coli* O157 outbreak occurred in 1999 at a county fair due to contaminated drinking water and involved 781 ill persons; 9% were hospitalized, HUS developed in 2%, and 2 died (26). The implicated water was from a temporary unregulated well at the fairground. Properly functioning water systems with adequate chlorine levels should protect against *E. coli* O157 contamination. Many U.S. households, however, receive municipal water that is not chlorinated. Further safeguards are therefore needed to ensure the safety of unchlorinated water systems and to ensure that chlorinated water systems are properly functioning. Educational efforts targeted at caretakers of young children should continue to help reduce contamination of recreational water areas by fecal matter (27,28).

Outbreaks associated with animal contact represent a newly recognized transmission route for *E. coli* O157 in the United States. Cattle hides may become contaminated from fecal matter. Persons touching cattle or surfaces in the cattle's environment may contaminate their hands with *E. coli* O157. If hands are not washed thoroughly after contact with cattle or their environments, the bacteria can infect these persons through a hand-to-mouth route. Recent strategies published to help reduce transmission of enteric pathogens from farm animals to children include informing the public about risk for transmission of enteric pathogens from farm animals to humans, separating eating facilities from animal contact areas, and providing adequate handwashing facilities (29).

The overall decreased HUS and case-fatality rates in the last 2 decades likely represent increased reporting of less clinically severe outbreaks, especially after *E. coli* O157 became a reportable disease. The high HUS rate found in swimming-associated outbreaks may be due partly to the higher proportion of young children involved and their vulnerability to development of HUS. The reason for the higher HUS rate found among ground beef–related outbreaks is unclear and may reflect reporting bias. Outbreaks occurring in residential facilities such as nursing homes had a particularly high case-fatality rate, which emphasizes the need for prevention efforts, both educational and regulatory, to lower the incidence of *E. coli* O157 infections in such facilities.

Since 1992, molecular subtyping of *E. coli* O157 by pulsed-field gel electrophoresis has improved early outbreak detection. PulseNet (30), the national network for comparing molecular subtypes of common foodborne bacterial pathogens, including *E. coli* O157 since 1997, has greatly assisted in both identifying outbreaks and linking apparently unrelated outbreaks. Continued molecular subtyping of *E. coli* O157 strains from both humans and the environment will assist in detecting outbreaks and allow for identification of multistate, geographically dispersed outbreaks due to contaminated commercial products (30).

Outbreak surveillance has several limitations. *E. coli* O157 outbreaks captured by CDC's surveillance system likely represent only a small proportion of outbreaks that occur. Many outbreaks go unrecognized, are classified as outbreaks of unknown etiology, and are not reported to local public health officials or CDC (31). Smaller outbreaks and outbreaks with unknown transmission routes and vehicles are less likely to be reported, and this summary likely under represents such outbreaks. Including patients with compatible clinical illness without culture confirmation is another limitation of outbreak surveillance. However, given the broad clinical spectrum of *E. coli* O157 infection, and the limited number of infected persons with culture-confirmed illness (5), such inclusion allows us to better assess the true public health impact of *E. coli* O157. In addition, outbreak reporting may not be uniform across time periods or states. Therefore, trends should be interpreted carefully, given the changing factors that may impact outbreak detection and reporting. The increased numbers of outbreaks reported since 1993 but with smaller sizes are likely due to increased awareness of disease, improved diagnostics, increased *E. coli* O157 testing, and improved outbreak detection through molecular subtyping.

Outbreak investigations, especially for emerging pathogens such as *E. coli* O157, are critical for better understanding these pathogens' epidemiology, which affect policy and behavior changes. While a summary of outbreaks cannot draw firm conclusions on disease trends, illustration of transmission routes, food vehicles, outbreak size, and clinical outcomes over time empowers public health officials, regulatory agencies, and health educators to target appropriate interventions and reevaluate current prevention strategies.
